# Pharmacological targeting of α3β4 nicotinic receptors improves peripheral insulin sensitivity in mice with diet-induced obesity

**DOI:** 10.1007/s00125-020-05117-4

**Published:** 2020-03-06

**Authors:** Sigrid Jall, Meri De Angelis, Anne-Marie Lundsgaard, Andreas M. Fritzen, Trine S. Nicolaisen, Anders B. Klein, Aaron Novikoff, Stephan Sachs, Erik A. Richter, Bente Kiens, Karl-Werner Schramm, Matthias H. Tschöp, Kerstin Stemmer, Christoffer Clemmensen, Timo D. Müller, Maximilian Kleinert

**Affiliations:** 1 Institute for Diabetes and Obesity, Helmholtz Diabetes Center at Helmholtz Zentrum München, Ingolstädter Landstraße 1, 85764 Neuherberg, Germany; 2 German Center for Diabetes Research (DZD), München-Neuherberg, Germany; 3 Division of Metabolic Diseases, TUM School of Medicine, Technische Universität München, Munich, Germany; 4 Molecular EXposomics (MEX) at Helmholtz Zentrum München, Neuherberg, Germany; 5 Section of Molecular Physiology, Department of Nutrition, Exercise and Sports, Faculty of Science, University of Copenhagen, Copenhagen, Denmark; 6 Novo Nordisk Foundation Center for Basic Metabolic Research, Faculty of Health and Medical Sciences, University of Copenhagen, Blegdamsvej 3B, DK-2200 Copenhagen N, Denmark; 7 Institute of Diabetes and Regeneration Research, Helmholtz Diabetes Center at Helmholtz Zentrum München, Neuherberg, Germany; 8 Department für Biowissenschaften, Wissenschaftszentrum Weihenstephan für Ernährung, Landnutzung und Umwelt, Technische Universität München, Freising, Germany; 9 Helmholtz Zentrum München, German Research Center for Environmental Health (GmbH), Munich-Neuherberg, Germany; 10 Department of Biology, University of Konstanz, Konstanz, Germany; 11 Department of Pharmacology, Experimental Therapy and Toxicology, Institute of Experimental and Clinical Pharmacology and Pharmacogenomics, Eberhard Karls University Hospitals and Clinics, Tübingen, Germany

**Keywords:** Catecholamine, Glucose metabolism, Glucose tolerance, Hyperglycaemia, Insulin sensitivity, Nicotinic acetylcholine receptor, Pharmacology

## Abstract

**Aims/hypothesis:**

Treatment with the α3β4 nicotinic acetylcholine receptor (nAChR) agonist, 1,1-dimethyl-4-phenylpiperazinium iodide (DMPP), improves glucose tolerance in diet-induced obese (DIO) mice, but the physiological and molecular mechanisms are unknown.

**Methods:**

DMPP (10 mg/kg body weight, s.c.) was administered either in a single injection (acute) or daily for up to 14 days (chronic) in DIO wild-type (WT) and *Chrnb4* knockout (KO) mice and glucose tolerance, tissue-specific tracer-based glucose metabolism, and insulin signalling were assessed.

**Results:**

In WT mice, but not in *Chrnb4* KO mice, single acute treatment with DMPP induced transient hyperglycaemia, which was accompanied by high plasma adrenaline (epinephrine) levels, upregulated hepatic gluconeogenic genes, and decreased hepatic glycogen content. In contrast to these acute effects, chronic DMPP treatment in WT mice elicited improvements in glucose tolerance already evident after three consecutive days of DMPP treatment. After seven days of DMPP treatment, glucose tolerance was markedly improved, also in comparison with mice that were pair-fed to DMPP-treated mice. The glycaemic benefit of chronic DMPP was absent in *Chrnb4* KO mice. Chronic DMPP increased insulin-stimulated glucose clearance into brown adipose tissue (+69%), heart (+93%), gastrocnemius muscle (+74%) and quadriceps muscle (+59%), with no effect in white adipose tissues. After chronic DMPP treatment, plasma adrenaline levels did not increase following an injection with DMPP. In glucose-stimulated skeletal muscle, we detected a decreased phosphorylation of the inhibitory Ser640 phosphorylation site on glycogen synthase and a congruent increase in glycogen accumulation following chronic DMPP treatment.

**Conclusions/interpretation:**

Our data suggest that DMPP acutely induces adrenaline release and hepatic glycogenolysis, while chronic DMPP-mediated activation of β4-containing nAChRs improves peripheral insulin sensitivity independently of changes in body weight via mechanisms that could involve increased non-oxidative glucose disposal into skeletal muscle.

**Electronic supplementary material:**

The online version of this article (10.1007/s00125-020-05117-4) contains peer-reviewed but unedited supplementary material, which is available to authorised users.

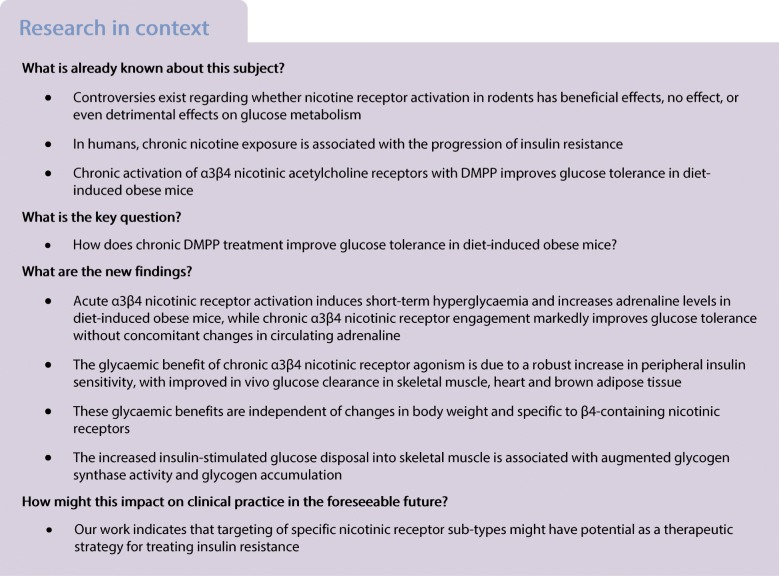



## Introduction

Obesity and associated metabolic disorders, such as type 2 diabetes, are major public health issues [1]. Considerable preclinical progress has been undertaken to tackle the obesity and type 2 diabetes pandemics, using different pharmacological strategies [2, 3]. Recently, the activation of central nicotine receptors has been suggested as a promising target to reduce food intake [4]. The nicotinic acetylcholine receptors (nAChRs) comprise several homo- or heteropentamers containing α subunits (CHRNA1-7 and CHRNA9-10) and/or β subunits (CHRNB1-4) [5]. Mineur et al have shown that pharmacological targeting of central nAChRs with nicotinic agonists can suppress food intake in mice [4]. In line with this, in humans, inhalation of nicotine-dense tobacco smoke is associated with a lower body weight, while smoking cessation is accompanied by body weight gain [6, 7]. However, inhalation of the broad nAChR-agonist nicotine with smoking is also associated with an increase in cancer risk [8, 9], non-alcoholic fatty liver disease [10], and peripheral insulin resistance [11] in humans.

We have recently shown that selective targeting of the α3β4 nAChRs with 1,1-dimethyl-4-phenylpiperazinium iodide (DMPP) reduces food intake and lowers body weight in diet-induced obese (DIO) mice [2]. In addition, we made the serendipitous observation that DMPP treatment for 7 days also robustly improved glucose tolerance. Even when administered at doses below the threshold needed to reduce body weight, DMPP ameliorated an impaired glucose tolerance [2]. This suggests that DMPP-mediated α3β4 nAChR engagement per se improves glucose tolerance; however, this has not been formally tested. In addition, the physiological and molecular mechanisms underpinning the glycaemic benefit of DMPP are unknown. For instance, it remains to be clarified whether DMPP improves glucose tolerance because of enhanced insulin secretion or because of effects on peripheral insulin sensitivity (or both). Furthermore, it is unknown whether DMPP-mediated improvements in glucose tolerance require functional α3β4 nAChRs. Therefore, we here assessed the glucometabolic effects of DMPP in ad libitum-fed DIO mice, in DIO mice pair-fed to DMPP-treated mice, and in knockout (KO) DIO mice that lack β4 nAChR (neuronal acetylcholine receptor subunit β-4 [CHRNB4], which is encoded by *Chrnb4*).

## Methods

### Mice for pharmacological studies

For wild-type (WT) mouse studies, male C57BL/6J mice were obtained from Janvier Labs (Le Genest Saint Isle, France). *Chrnb4* KO mice were generated as described previously [12]. Heterozygous *Chrnb4* KO mice on a C57BL/6J background were kindly provided by U. Maskos (Institut Pasteur, Paris, France) and bred in-house at the Helmholtz Zentrum München, Neuherberg, Germany. Homozygous *Chrnb4* KO and WT mice were used to generate colonies of *Chrnb4* KO and WT mice. All mice were switched from a regular chow diet to a high-fat, high-sucrose diet (D12331; Research Diets, New Brunswick, NJ, USA) at an age of 8 weeks, with ad libitum access to water and diet. Mice were maintained at 23°C ambient temperature under specific pathogen-free conditions at constant humidity and on a 12 h light–dark cycle.

### Pharmacological intervention studies

Interventions were performed in DIO mice weighing ~50 g. DIO mice are used as a model for obesity and insulin resistance associated with the human metabolic syndrome, an established risk factor for development of type 2 diabetes [13]. Mice were randomly assigned to pharmacological treatment groups based on body weight or ad libitum-fed blood glucose. For the pair-feeding study, daily food of pair-fed mice was matched to food intake of DMPP-treated mice. The experimenters were not blinded to the intervention groups. DMPP (D5891, Sigma-Aldrich, Munich, Germany) was administered in saline (NaCl 0.9%) at 10 mg/kg body weight with the vehicle control group receiving 1% DMSO in saline. Compounds were administered s.c. at a volume of 5 μl/g body weight. In the acute set-up, food was removed and ad libitum blood glucose was determined at 07:00 hours in blood sampled from the tail vein and measured using handheld glucometers (Abbott, Wiesbaden, Germany). Blood for insulin measurements was collected at time points as indicated in the figures by bleeding mice from the tail vein into EDTA-coated microvette tubes (Sarstedt, Nümbrecht, Germany). For assessment of plasma catecholamines after acute DMPP, blood was collected into EDTA-coated microvette tubes (Sarstedt) 80 min after DMPP injection. For chronic studies, unless stated otherwise, DMPP was injected s.c. daily in the late afternoon. For glucose tolerance tests, mice were fasted for 6 h (starting at 07:30 hours) and received i.p. injections of glucose at 1.75 g/kg body weight. In the chronic pair-feeding and *Chrnb4* KO studies, glucose tolerance tests were performed on day 7. On day 10 (i.e. after 10 days of daily DMPP injections) mice were fasted for 6 h (starting at 07:30 hours), glucose (1.75 g/kg body weight) was i.p. injected, and after 30 min mice were euthanised by cervical dislocation and blood and tissues were quickly collected.

For measurement of plasma catecholamines after 7 days of daily DMPP injections, blood was collected 80 min after last compound injections (on day 8) into EDTA-coated microvette tubes (Sarstedt). In the chronic studies lasting for 14 days, mice were injected with vehicle or DMPP and fasted 2 h prior to killing. All animal experimentations were approved and conducted in accordance to the Danish Animal Experimentation Inspectorate and Animal Ethics Committee of the Government of Upper Bavaria, Germany.

### Plasma variables

The collected blood was immediately kept on ice and centrifuged for 10 min at 3000 *g* and 4°C. Plasma was stored at −20°C until further analysis. Plasma insulin levels were analysed using a commercially available ELISA kit (Alpco Diagnostics, Salem, NH, USA) following the manufacturers’ instructions. Plasma catecholamines were analysed and detected with an HPLC system coupled with an electrochemical detector (EcD) as described previously [14]. The sample clean-up was performed according to the protocol described by Recipe (Recipe, Munich, Germany). The Recipe ClinRep complete kit contains all necessary chemicals and materials for the extraction. The limited amount of plasma per sample necessitated some modifications from the standard protocol. Therefore, 30–40 μl of plasma was diluted with 40 μl of water and 10 μl of internal standard was added. Upon vigorous mixing, the samples were charged on the sample preparation column. The column was shaken for 10 min and the solvent was removed on a vacuum manifold. The column was washed three times with 1 ml washing solution to remove interfering components. After drying the column, the elution reagent was added (140 μl). The catecholamines were eluted from the extraction column via centrifugation and 20 μl of the eluate was injected into the HPLC-EcD system.

### Gene expression analysis

For tissue collection, mice were either euthanised using CO_2_ or killed by cervical dislocation. Tissues were extracted and immediately kept on dry ice or liquid nitrogen and stored at −80°C until further analysis. For gene expression analysis, liver RNA was isolated using the TRIzol-based RNeasy Kit (Qiagen, Hilden, Germany) according to the manufacturers’ instructions. cDNA was synthesised from total RNA using QuantiTect Reverse Transcription Kit (Qiagen). Gene expression profiles were assessed in the liver with the quantitative real-time PCR technique using SYBR green (Thermo Fisher Scientific, Erlangen, Germany). Relative gene expression was normalised to the reference gene *Hprt*. Primer sequences used are listed in alphabetical order in electronic supplementary material (ESM) Table 1.

### Tissue glycogen

Hepatic and muscle glycogen was measured in 20–40 mg of tissue using a commercially available kit (Biovision, Milpitas, CA, USA) following the manufacturers’ instructions.

### Western blot analyses

Approximately 25 mg of quadriceps muscle was homogenised (Tissue Lyzer II, Qiagen) in ice-cold buffer as described previously [15]. Homogenates were rotated end-over-end for 1 h and lysate supernatants were collected by centrifuging for 20 min at 16,000 *g* and 4°C. Protein concentrations were assessed using the bicinchoninic acid method (Pierce Biotechnology, Rockford, IL, USA). Samples were heated (96°C) in Laemmli buffer before being subjected to SDS-PAGE and semi-dry blotting. The primary antibodies used were from Alpha Diagnostics (San Antonio, TX, USA) (hexokinase II #HXK23-A [1:1000], RRID AB_2117140); Cell Signaling Technology (Danvers, MA, USA) (Akt2 #3063 [1:1000], RRID AB_2225186; p-Akt Thr308 #9275 [1:1000], RRID AB_329828; p-Akt Ser473 #9271 [1:1000], RRID AB_329825; phospho Akt substrate [PAS] #9611 [1:500], RRID AB_330302; p-TBC1 domain family member [TBC1D] 1 Thr596 #6927 [1:1000], RRID AB_10828720); Thermo Fisher Scientific (Waltham, MA, USA) (GLUT4 #PA1-1065 [1:1000], RRID AB_2191454); Millipore (Burlington, MA, USA) (TBC1D4 #07-741 [1:1000], RRID AB_492639). TBC1D1, p-glycogen synthase Ser640, and glycogen synthase were kindly donated by G. Hardie, University of Dundee, UK. Secondary antibodies were from Jackson ImmunoResearch (Ely, UK) (1:3000). Membranes were probed with enhanced chemiluminescence (ECL^+^; Amersham Biosciences, Piscataway, NJ, USA) and immune complexes were visualised using ChemiDoc MP Imaging System (Bio-Rad Laboratories, Hercules, CA, USA). Signals were quantified (Image Lab, Bio-Rad Laboratories) and expressed as arbitrary units.

### In vivo 2-deoxy-glucose clearance and glucose incorporation into glycogen

For assessment of glucose-induced glucose clearance and glucose incorporation into glycogen, glucose at 1.75 g/kg body weight was i.p. injected at 10 μl/g body weight together with ^3^H-labelled 2-deoxy-glucose (^3^H-2-DG) (2.22 MBq/ml) and D-6-^14^C-labelled glucose (0.185 MBq/ml), respectively, to mice treated with DMPP daily for 8 days and fasted for 6 h. Blood glucose was measured at the indicated time points using a handheld glucometer (Arseus Medical, Bornem, Belgium). For analysis of ^3^H-2-DG clearance in indicated tissues, plasma ^3^H activity was measured at 10, 20, and 40 min in 5 μl of blood by scintillation counting and systemic ^3^H-2-DG exposure estimated by the trapezoidal method. A 25 mg sample of each tissue was used to determine the accumulation of ^3^H-2-DG-6-phosphate (^3^H-2-DG-6-P) by the precipitation method [16]. Glucose clearance was calculated by dividing tissue ^3^H-2-DG-6-P counts by systemic ^3^H-2-DG exposure [17]. For muscle glucose incorporation into glycogen, ~15 mg of gastrocnemius muscle was weighed out, mixed with 1 ml of 1 mol/l NaOH, and boiled at 99.5°C for 30 min. To facilitate the precipitation of glycogen, 170 μl of glycogen (4 mg/ml, G-8876, Sigma-Aldrich) was added and samples mixed. Ice-cold 96% ethanol (800 μl) was added, samples mixed, and stored at −20°C overnight. Samples were centrifuged at 1200 *g* for 15 min, the ethanol was removed, and the pellet was re-suspended in 2 ml of ice-cold 96% ethanol. After another centrifugation at 1200 *g* for 15 min, ethanol was removed and 550 μl H_2_O was added to dilute the pellet; 525 μl of the sample was added to 3 ml of scintillation fluid, vigorously mixed, and analysed by scintillation counting for ^14^C (Packard TriCarb 2900TR, Perkin-Elmer, Boston, MA, USA). Glucose incorporation into glycogen was expressed as ^14^C glucose counts in the glycogen precipitated.

### Statistics

Data were analysed in GraphPad Prism (versions 6 and 8; GraphPad Software, San Diego, CA, USA). The types of statistical tests performed are outlined in the figure legends. A *p* value ≤0.05 was considered statistically significant. All results are presented as mean ± SEM.

## Results

### DMPP has acute hyperglycaemic effects, but exerts glycaemic benefits after 3 days of daily administration in DIO mice

**Fig. 1:**
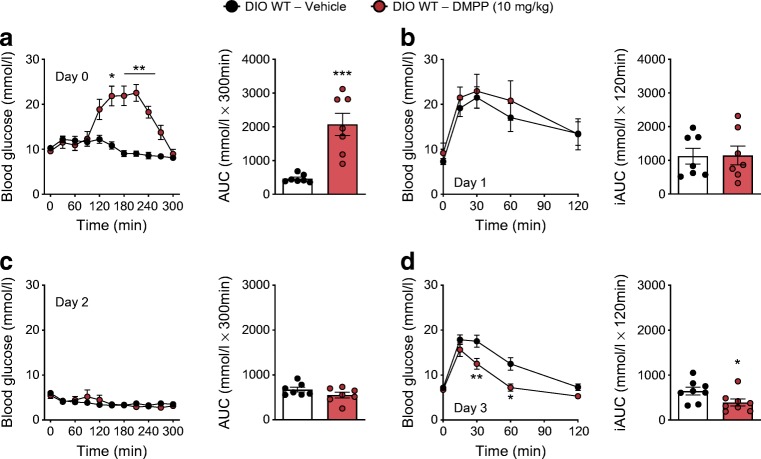
DMPP acutely elicits hyperglycaemia, while chronically it improves glucose tolerance. (**a**) Effect of first injection (i.e. day 0) of DMPP (10 mg/kg) or vehicle injected at time point 0 min on blood glucose excursion with AUC in DIO WT mice. (**b**) Glucose tolerance with incremental AUC (iAUC) determined 24 h after the first injection (i.e. day 1) of DMPP or vehicle. (**c**) Effect of the third injection (i.e. day 2) of DMPP or vehicle on blood glucose excursion and AUC. (**d**) Glucose tolerance with iAUC determined 18 h after the third daily injection (i.e. day 3) of DMPP or vehicle. All data are presented as mean ± SEM (*n* = 7–8). Data in line graphs were assessed by two-way repeated measures ANOVA (time × drug) with a subsequent Bonferroni post hoc test. Data in bar graphs were probed with two-tailed Student’s *t* tests, comparing the means of vehicle and DMPP; *p ≤ 0.05, **p ≤ 0.01, ***p ≤ 0.001 compared with vehicle

A single s.c. administration of DMPP (10 mg/kg body weight) increased ad libitum*-*fed blood glucose by ~200% in comparison with vehicle in DIO mice, with a peak at ~2 h after treatment and a return to baseline after 5 h (Fig. 1a). This short-term hyperglycaemic effect of DMPP was absent in mice that globally lack the β4 subunit of the α3β4 nAChRs (*Chrnb4* KO mice) (ESM Fig. 1a, b). Despite its short-term hyperglycaemic effect, a single treatment with DMPP did not affect glucose tolerance in DIO mice the following day (day 1) (Fig. 1b). After two days of daily DMPP administration, injection of DMPP had no effect on ad libitum*-*fed blood glucose (Fig. 1c) and already after the third DMPP injection (day 3), glucose tolerance in DIO mice was significantly improved (Fig. 1d).

### DMPP acutely increases circulating adrenaline and lowers hepatic glycogen content

**Fig. 2:**
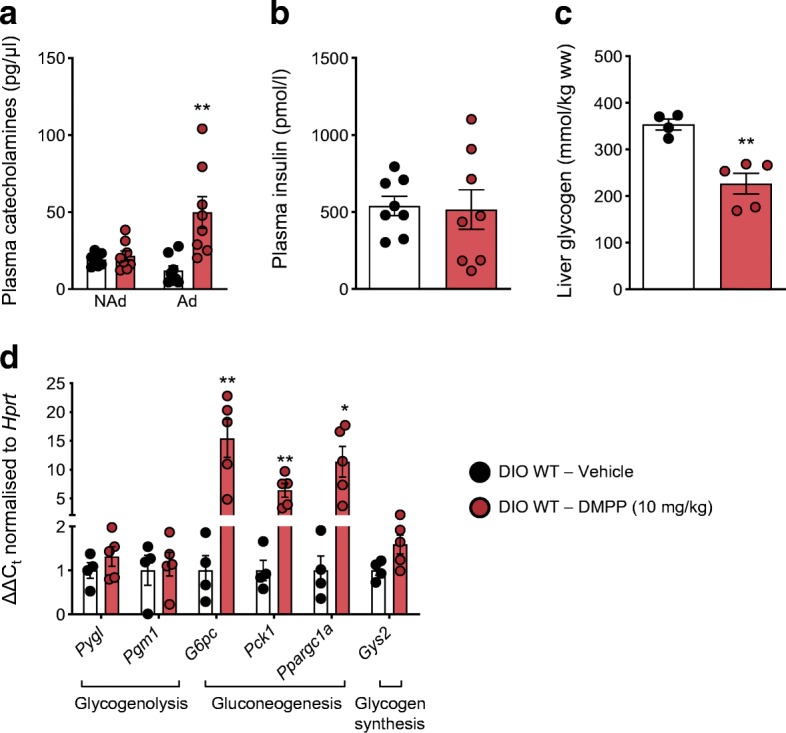
Acute DMPP increases circulating adrenaline and induces hepatic gluconeogenesis and glycogenolysis. (**a**–**d**) Effect of first injection of DMPP (10 mg/kg) or vehicle in DIO WT mice on (**a**) plasma noradrenaline (NAd) and adrenaline (Ad) concentrations determined in blood collected 80 min after vehicle or DMPP was injected (*n* = 8); on (**b**) plasma insulin concentrations in blood collected 80 min after vehicle or DMPP was injected (*n* = 8); on (**c**) liver glycogen at 150 min after vehicle or DMPP injection (*n* = 4–5); and on (**d**) expression of indicated genes in the liver at 150 min after vehicle or DMPP injection (*n* = 4–5). All data are presented as mean ± SEM. Data were probed with two-tailed Student’s *t* tests, comparing the means of vehicle and DMPP; *p ≤ 0.05, **p ≤ 0.01 compared with vehicle. ww, wet weight

The transient hyperglycaemia observed in DIO mice after the first single DMPP injection was associated with a fourfold increase in plasma adrenaline 80 min after injection (*p* ≤ 0.01), while circulating noradrenaline (Fig. 2a) and insulin (Fig. 2b) were unaltered. Adrenaline is known to increase hepatic glucose output [18] and, in agreement, hepatic glycogen content in DMPP-treated mice was 36% lower 150 min after injection compared with vehicle (Fig. 2c; *p* ≤ 0.01). Liver glycogen breakdown was accompanied by an increase in the expression of hepatic genes involved in gluconeogenesis 150 min after administration (Fig. 2d). After 7 days of daily DMPP treatment (chronic), DMPP injection on day 8 did not have effects on circulating adrenaline levels (ESM Fig. 2b) and DMPP administration did not induce changes in hepatic gluconeogenic gene expression after 14 days of treatment (ESM Fig. 2c).

### Chronic DMPP improves glucose tolerance independent of changes in body weight, via CHRNB4, and without increasing insulin secretion

**Fig. 3:**
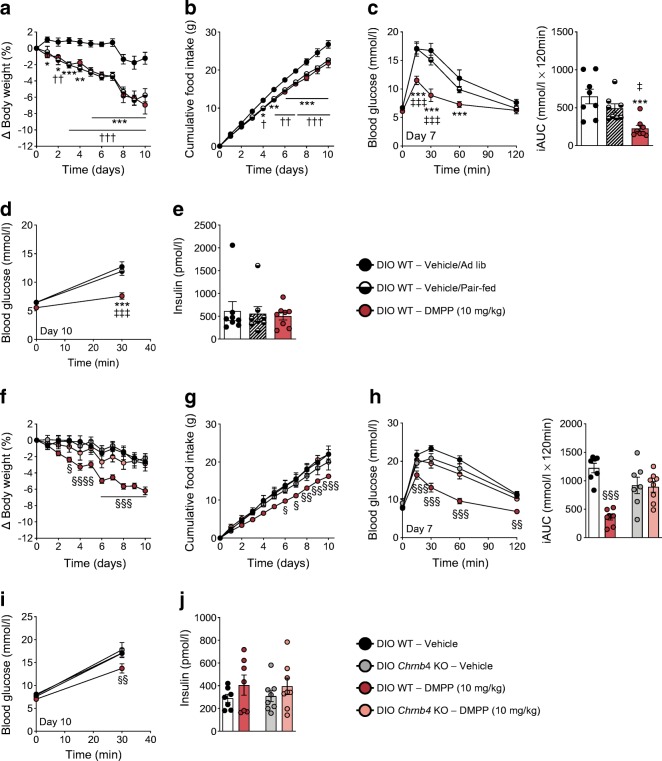
Chronic DMPP improves glucose tolerance independent of body weight loss and specifically via CHRNB4. (**a**, **b**) Body weight loss (in %) and cumulative food intake (in g) of DMPP-treated and vehicle-treated DIO WT mice with either ad libitum (Ad lib) food access or pair-fed to mice receiving 10 mg/kg DMPP for 10 days. (**c**) Glucose tolerance with incremental AUC (iAUC) conducted 18 h after the seventh injection of daily vehicle or DMPP (i.e. day 7). (**d**) Plasma glucose (at 0 and 30 min) and (**e**) plasma insulin at 30 min after i.p. injection of glucose (injected at time point 0 min), 18 h after the tenth injection of daily vehicle or DMPP (i.e. day 10). (**f**, **g**) Body weight loss (in %) and cumulative food intake (in g) in DIO WT or *Chrnb4* KO mice receiving vehicle or DMPP. (**h**) Glucose tolerance with respective iAUC 18 h after seventh injection of daily vehicle or DMPP. (**i**) Plasma glucose (0 and 30 min) and (**j**) plasma insulin at 30 min after i.p. injection of glucose (injected at time point 0 min) 18 h after tenth injection of daily vehicle or DMPP. All data are presented as mean ± SEM; (*n* = 7–8). Data in line graphs were assessed by two-way repeated measures ANOVA (time × drug) within genotypes with a subsequent Bonferroni post hoc test. Data in (**e**) and bar graph in (**c**) were assessed by one-way ANOVA, and with a subsequent Bonferroni post hoc test (for **c**). Data in (**j**) and in the bar graph in (**h**) were assessed with two-tailed Student’s *t* tests within the genotypes. *p ≤ 0.05, **p ≤ 0.01, ***p ≤ 0.001 for vehicle compared with DMPP; ^†^*p* ≤ 0.05, ^††^*p* ≤ 0.01, ^†††^*p* ≤ 0.001 for vehicle compared with pair-fed; ^‡^*p* ≤ 0.05, ^‡‡‡^*p* ≤ 0.001 for pair-fed compared with DMPP; ^§^*p* ≤ 0.05, ^§§^*p* ≤ 0.01, ^§§§^*p* ≤ 0.001 for vehicle compared with DMPP within WT

Chronic treatment with DMPP improves glucose tolerance in DIO mice [2]. However, it is unknown whether this: (1) occurs independently of changes in body weight; (2) requires functional α3β4 nAChRs; and (3) is the consequence of increased insulin secretion or improved peripheral insulin sensitivity. We therefore assessed glucose tolerance in vehicle- and DMPP-treated mice, as well as in vehicle-treated mice that were pair-fed to the DMPP group. Ten days of DMPP administration induced a significant reduction in food intake and body weight compared with vehicle-treated mice (Fig. 3a, b). Pair-fed vehicle-treated mice lost a similar amount of body weight as the DMPP-treated mice (~5%). Fasted blood glucose levels on day 7 and on day 10 were not different among the three groups (Fig. 3c, d). However, glucose tolerance was robustly improved in DMPP-treated mice compared with both vehicle-treated and pair-fed vehicle-treated mice (Fig. 3c, d).

Second, we utilised *Chrnb4* KO mice to investigate whether glycaemic benefits of DMPP require β4-containing nAChRs. In DIO WT mice, but not DIO *Chrnb4* KO mice, daily administration of DMPP for 10 days led to significantly lower body weight and food intake (Fig. 3f, g). Genotype or DMPP treatment did not affect fasted blood glucose levels on day 7 and on day 10 (Fig. 3h, i), but DMPP significantly improved glucose tolerance in DIO WT mice, yet failed to do so in *Chrnb4* KO mice (Fig. 3h, i).

Third, we measured plasma insulin levels 30 min after i.p. glucose injection and detected no differences in circulating insulin in DMPP-treated mice compared with control mice (Fig. 3e, j), suggesting that improvements in glucose tolerance are not mediated by augmented insulin secretion.

### Chronic DMPP increases glucose uptake in brown adipose tissue and striated muscles

**Fig. 4:**
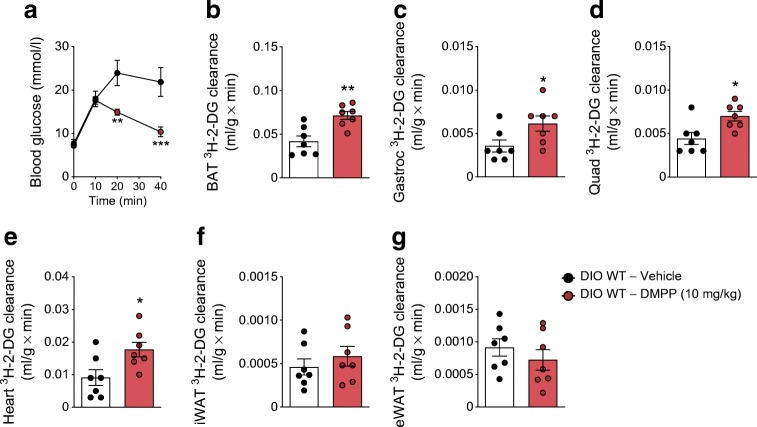
Chronic DMPP selectively increases glucose clearance in the BAT and the muscles. (**a**) Glucose excursion after injection with glucose 18 h after the eighth injection of daily DMPP (10 mg/kg) or vehicle in DIO WT mice. Glucose clearance into the (**b**) BAT, (**c**) gastrocnemius (Gastroc) muscle, (**d**) quadriceps (Quad) muscle, (**e**) heart, (**f**) iWAT, and (**g**) eWAT. All data are presented as mean ± SEM (*n* = 7). Data in line graph in (**a**) were assessed by two-way repeated measures ANOVA (time × drug) with a subsequent Bonferroni post hoc test. All other data (**b–g**) were probed with two-tailed Student’s *t* tests, comparing the means of vehicle and DMPP. *p ≤ 0.05, **p ≤ 0.01, ***p ≤ 0.001 for the effect of drug at the indicated time point or, in bar graphs, compared with vehicle

After 8 days of daily DMPP administration, improved glucose tolerance (Fig. 4a) was associated with increased glucose disposal into brown adipose tissue (BAT) (+69%), gastrocnemius muscle (+74%), quadriceps muscle (+59%), and heart (+93%) with no effects in inguinal or epididymal white adipose tissue (iWAT and eWAT) (Fig. 4b–g). In accordance with what we observed above, chronic DMPP did not increase glucose-stimulated plasma insulin levels (ESM Fig. 2a).

### DMPP has no effect on Akt signalling, but increases glycogen content in skeletal muscle

**Fig. 5:**
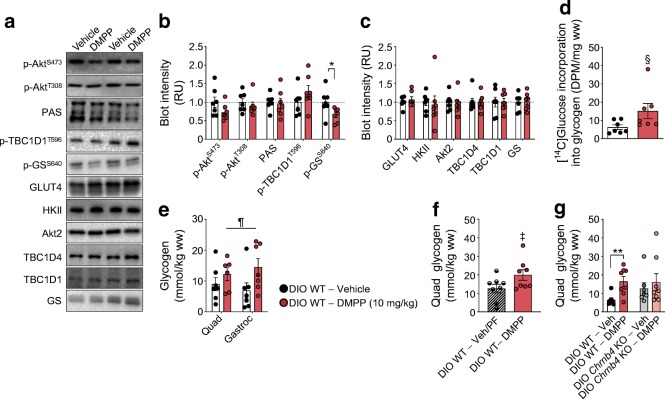
DMPP increases non-oxidative glucose disposal in skeletal muscle. (**a–c**) Representative western blots and quantification of indicated protein phosphorylation residues or total proteins, relative to total protein, in quadriceps muscle from DIO WT mice treated as described in Fig. 4. (**d**, **e**) [^14^C]Glucose incorporation into glycogen and glycogen content in quadriceps (Quad) and gastrocnemius (Gastroc) muscles from DIO WT mice treated as described in Fig. 4. (**f**) Quadriceps muscle glycogen from vehicle-treated pair-fed (PF) and DMPP-treated mice (10 mg/kg); and (**g**) from vehicle- or DMPP-treated DIO WT and *Chrnb4* KO mice (10 mg/kg). The key next to (**e**) applies to (**b**–**e**). All data are presented as mean ± SEM. For (**a**–**e**) *n* = 7, except for DMPP-treated quadriceps muscle in (**e**) for which *n* = 6, because of insufficient material for one of the samples; (**f**) *n* = 8; (**g**) *n* = 7–8. Differences were probed with two-tailed Student’s *t* tests for (**b**, **c**, **d**, **f**, **g**) comparing the means of vehicle and DMPP. Data in (**e**) were analysed with two-way repeated measures ANOVA (muscle × drug). *p ≤ 0.05, **p ≤ 0.01 for DMPP compared with vehicle; ^§^*p* = 0.064 for DMPP compared with vehicle; ^**¶**^*p* ≤ 0.05 main effect of DMPP; ^‡^*p* ≤ 0.05 vehicle-treated pair-fed compared with DMPP. DPM, disintegrations/min; GS, glycogen synthase; HKII, hexokinase II; RU, relative units; ww, wet weight

Given the quantitative importance of skeletal muscle in whole-body postprandial glucose disposal [19, 20], we investigated canonical insulin signalling in in vivo glucose-stimulated quadriceps muscle after 8 days of daily DMPP treatment. Insulin-responsive signalling proteins Akt, TBC1D1, and TBC1D4 displayed similar phosphorylation states in DMPP- and vehicle-treated mice (Fig. 5a–c) and the protein abundance of GLUT4 was also comparable between the two groups (Fig. 5a, c). Notably, however, we observed a reduction in the Ser640 phosphorylation site on glycogen synthase in DMPP-treated mice (Fig. 5a, b). Glycogen synthase Ser640 phosphorylation suppresses glycogen synthase activity [21, 22], suggesting an increased glycogen synthase activity after chronic DMPP treatment. Congruent with these data, we observed apparently augmented incorporation of labelled glucose into glycogen, although this failed to reach statistical significance (Fig. 5d, *p* = 0.064) and significantly increased skeletal muscle glycogen content in DMPP-treated mice, also compared with pair-fed mice with a similar loss in body weight (Fig. 5e, f, *p* ≤ 0.05). Conversely, DMPP failed to increase muscle glycogen in *Chrnb4* KO mice (Fig. 5g).

## Discussion

We here show that DMPP engages β4-containing nAChRs to improve glucose tolerance in obese mice after only a few days of administration. This glycaemic benefit of DMPP occurs independent of changes in body weight and by enhancing peripheral insulin sensitivity. DMPP treatment increased in vivo insulin-stimulated glucose clearance selectively into BAT, skeletal muscle, and the heart, but not into WAT. In skeletal muscle, canonical insulin signalling for glucose uptake was not altered by DMPP, while inhibitory glycogen synthase phosphorylation was decreased and muscle glycogen content increased.

DMPP has previously been shown to lower body weight and improve glucose tolerance [2], yet it was unclear whether the glycaemic benefit is secondary to the decrease in body weight. By pair-feeding mice to DMPP-treated mice we show that DMPP lowers body weight primarily by inhibiting food intake, as the pair-fed vehicle-treated and DMPP-treated mice lost similar amounts of body weight. In agreement, it has previously been shown that DMPP does not increase energy expenditure in DIO WT mice during three days of daily administration [2]. Most importantly, we demonstrate that DMPP improves glucose tolerance via mechanisms independent from the moderate decrease in body weight. Notably, in contrast to DMPP-treated mice, the ~5% body weight loss in pair-fed mice was not sufficient to ameliorate the diet-induced glucose intolerance. To determine whether these pronounced anorectic and glycaemic effects of DMPP depend on nicotinic receptor signalling, we assessed chronic DMPP in DIO *Chrnb4* WT and KO mice and found that both require intact β4-containing nAChRs. Although this does not prove that the effects are mediated solely by α3β4 nAChRs, these data emphasise an important degree of specificity of DMPP, especially in contrast to the broad nAChR-agonist nicotine [23–26].

Subsequently, we aimed to understand how DMPP improves glucose tolerance, by assessing glucose clearance measurements with radioactive tracers. This demonstrated that DMPP promotes glucose disposal into selective peripheral tissues. There are several indications that this effect, as well as the improvements in glucose tolerance, are not due to increased insulin secretion. First, plasma insulin was not higher 30 or 40 min after glucose injection in DMPP-treated mice. It remains possible that earlier differences in insulin levels were missed. Second, proximal insulin signalling in the form of Akt phosphorylation and downstream Akt activity proxies were similar between vehicle- and DMPP-treated muscles, suggesting that the tissue was exposed to comparable amounts of circulating insulin. Finally, if higher insulin levels/secretion were behind the enhanced glucose disposal, we would have expected augmented glucose disposal into all insulin-responsive tissues, but this was not the case. Instead, DMPP-treated mice exhibited increased glucose-mediated glucose clearance selectively into muscle, heart, and BAT, but not into subcutaneous or visceral WAT. The increase in BAT glucose clearance might be related to the previous observation that DMPP selectively induced protein expression of uncoupling protein 1 (UCP1) in BAT, but not in the iWAT of mice housed at thermoneutrality [2]. Of particular translational relevance could be the increase in muscle glucose uptake as the skeletal muscle comprises ∼40% of total body mass and is the key tissue for postprandial glucose disposal in humans [20]. Chronic DMPP treatment decreased Ser640 phosphorylation of glycogen synthase in muscle. In general, increased phosphorylation suppresses glycogen synthase activity, whereas a decrease in phosphorylation increases glycogen synthase activity [27, 28]. The Ser640 site has been proposed to play an important role in the regulation of glycogen synthase [21, 22, 29] and thus our data indicate that increased glycogen synthase activity with DMPP treatment augments non-oxidative glucose disposal in muscle. In support of this notion, muscle glycogen content was increased and glucose incorporation into glycogen in chronic DMPP-treated DIO WT mice was augmented; however this difference did not reach statistical significance. It remains to be clarified how glycogen synthase phosphorylation is modified by DMPP. The Akt–glycogen synthase kinase 3 (GSK3) signalling axis is known to regulate glycogen synthase phosphorylation, including the Ser640 residue [22, 30, 31]. In the presence of insulin, Akt inhibits GSK3 activity towards glycogen synthase, resulting in less phosphorylation of glycogen synthase to promote glycogen storage [32, 33]. However, since chronic DMPP did not alter Akt activity, other pathways are likely to be responsible for the decreased glycogen synthase phosphorylation in skeletal muscle. A potential signalling pathway could involve protein phosphatase 1, which can dephosphorylate glycogen synthase [34, 35].

The glycaemic benefit of chronic DMPP administration is counterintuitive to the transient hyperglycaemic episode observed following a single DMPP injection. However, there is precedence for differing acute vs chronic effects. Glucagon, for example, is a catabolic hormone that functions as a potent gluconeogenic agent acutely inducing hyperglycaemia, yet prolonged treatment with glucagon in DIO mice can result in glycaemic benefit [36]. Our data suggest that the acute hyperglycaemic effect of DMPP is mediated through a β4 nAChR-induced release of adrenaline. Notably, α3β4 nAChRs are expressed in adrenaline-releasing chromaffin cells of the adrenal medulla [37–39]. Adrenaline is known to trigger hepatic gluconeogenesis and glycogenolysis [40, 41]. In agreement, we found that acute DMPP administration in DIO WT mice potently induced hepatic gluconeogenic gene expression and that liver glycogen stores were reduced. Vu et al previously observed similar hyperglycaemic outcomes after treatment of lean mice with nicotine [42]. Of note, while DMPP only elicited increases in adrenaline secretion, nicotine increased both plasma adrenaline and noradrenaline [42]. In anaesthetised dogs, infusion of noradrenaline but not adrenaline induced marked insulin secretion under high glucose conditions [43]. This might explain why acute nicotine treatment raised insulin levels [42] when we observed no difference in insulin after DMPP injection. In the context of potential adverse cardiovascular effects, the rise in adrenaline upon acute DMPP warrants clarification. Notably, with repeated daily DMPP injections, the increase in plasma adrenaline as well as the induction of hepatic gluconeogenic genes was absent in the present study. In contrast, chronic nicotine treatment in lean mice resulted in elevated plasma adrenaline, reduced circulating noradrenaline, and a reduction of hepatic *Pck1* and *G6pc* mRNA levels [42]. We previously reported that lower doses of DMPP are sufficient to improve glucose tolerance in DIO mice [2]. It may thus be possible to avoid the early rise in adrenaline altogether by gradually increasing the DMPP dose to 10 mg/kg over several days, although that remains to be formally tested.

In summary, we report a temporal dichotomy in the glycaemic effects of DMPP. Acute hyperglycaemic effects are eclipsed by glycaemic benefits following chronic treatment, which are driven by a robust improvement in peripheral insulin sensitivity and an enhanced non-oxidative glucose disposal in skeletal muscle of DIO mice. Our findings advance the understanding of how nicotine receptor activation regulates glucose tolerance. Deciphering the exact molecular mechanisms of DMPP in the future could reveal novel treatment strategies for diabetes. We propose a novel drug candidate for the treatment of type 2 diabetes as the selective targeting of α3β4 nAChRs with DMPP markedly improved glucose tolerance by increasing glucose uptake in the BAT, heart, and the skeletal muscle, which accounts for 60–70% of postprandial glucose disposal [44].

## Electronic supplementary material

ESM (PDF 288 kb)

## Data Availability

The authors declare that all data supporting the findings of this investigation are available within the article, its supplementary information and from the corresponding authors upon reasonable request.

## Abbreviations

BAT: Brown adipose tissue
CHRNB4: Neuronal acetylcholine receptor subunit β-4
DIO: Diet-induced obese
DMPP: 1,1-Dimethyl-4-phenylpiperazinium iodide
eWAT: Epididymal white adipose tissue
GSK3: Glycogen synthase kinase 3
^3^H-2-DG: ^3^H-labelled 2-deoxy-glucose
^3^H-2-DG-6-P: ^3^H-2-DG-6-phosphate
iWAT: Inguinal white adipose tissue
KO: Knockout
nAChR: Nicotinic acetylcholine receptor
PAS: Phospho Akt substrate
TBC1D: TBC1 domain family member
WAT: White adipose tissue
WT: Wild-type
