# Supplementary material for: Pharmacological targeting of α3β4 nicotinic receptors improves peripheral insulin sensitivity in mice with diet-induced obesity
Source: Diabetologia. 2020 Mar 6;63(6):1236–47. doi: 10.1007/s00125-020-05117-4 (PMC7228898; doi:10.1007/s00125-020-05117-4)
Supplement: Supplementary file 1 — (PDF 288 kb) [file 125_2020_5117_MOESM1_ESM.pdf]

ESM Table 1 Sequences of primers

Gene	5’ – 3’ primer	5’ – 3’ primer
G6pc	CTGTCTGTCCCGGATCTACC	GCGCGAAACCAAACAAGAAG
Gys2	CGCTCCTTGTCGGTGACATC	CATCGGCTGTCGTTTTGGC
Hprt	AAGCTTGCTGGTGAAAAGGA	TTGCGCTCATCTTAGGCTTT
Pck1	CTGCATAACGGTCTGGACTTC	CAGCAAACTCCCGTACTCC
Pgm1	CCAAAATCTTGCGGGCCATA	CCAGAACAAAGGGACAGCAC
Ppargc1a	AGCCGTGACCACTGACAACGAG	GCTGCATGGTTCTGAGTGCTAAG
Pygl	TACATTCAGGCTGTGCTGGA	AAGGCATCAAACACGGTTCC

ESM Fig. 1

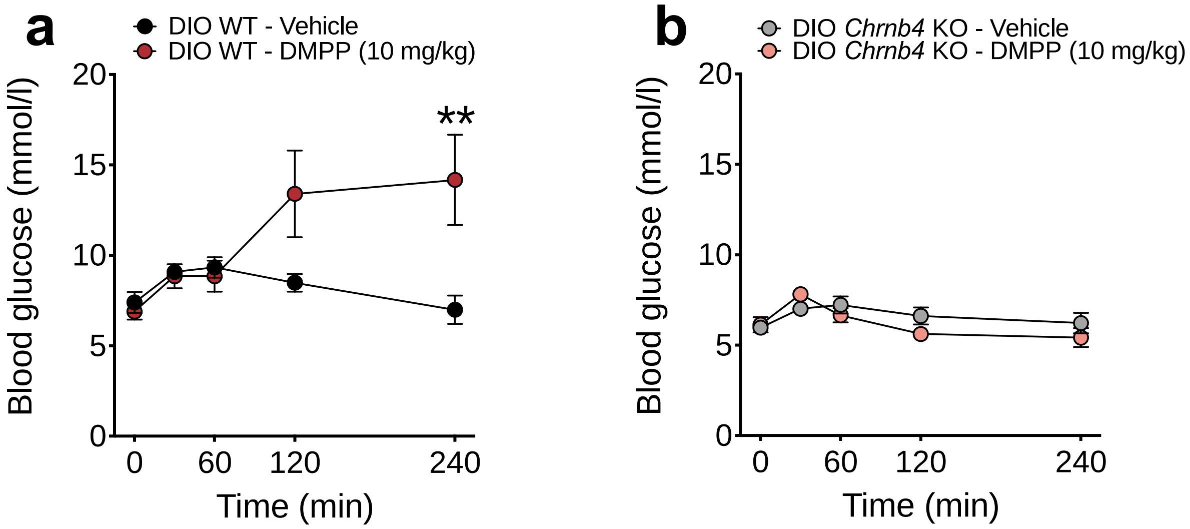

ESM Fig. 1 DMPP elicits hyperglycemia specifically via CHRNB4. (**a, b**) Effect of first injection of DMPP (10 mg/kg) or vehicle injected at time point 0 min on blood glucose in DIO WT and *Chrnb4* KO mice (n = 6-8). Data are means ± SEM. Data were assessed by two-way repeated measures ANOVA (time × drug) with a subsequent Bonferroni post hoc test. ***p*≤0.01 compared to vehicle at 240 min.

ESM Fig. 2

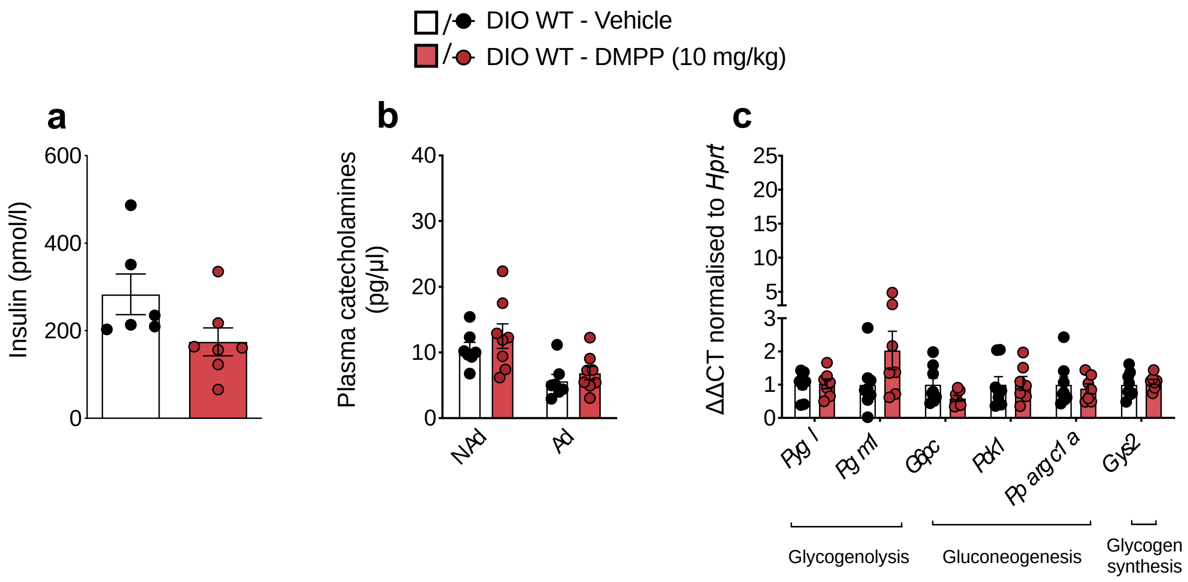

ESM Fig. 2 Glucose-stimulated insulin secretion, plasma catecholamines, and gluconeogenic gene expression after chronic DMPP. (**a**) Plasma insulin 40 min after glucose injection (1.75 g/kg body weight i.p.) in DIO mice chronically treated with DMPP (10 mg/kg) or vehicle for 8 days. (**b**) Plasma noradrenaline (NAd) and adrenaline (Ad) in chronically DMPP- or vehicle-treated DIO mice (day 8) 80 min after compound (DMPP or vehicle) injections. (**c**) Expression of indicated genes in the liver after 14 days of daily injections of vehicle or DMPP in DIO WT mice with liver dissection 2 hours after the last compound injection on day 14. Data are means ± SEM (n = 6-8). Differences were probed with two-tailed Student's *t*-tests comparing the means of vehicle and DMPP.
